# Infant body composition in a randomised trial of a maternal nutritional supplement during preconception and pregnancy

**DOI:** 10.1007/s12519-025-00900-y

**Published:** 2025-05-14

**Authors:** Jaz Lyons-Reid, José G. B. Derraik, Leigh C. Ward, Timothy Kenealy, Benjamin B. Albert, Jose M. Ramos Nieves, Cathriona R. Monnard, Mya Thway-Tint, Heidi Nield, Sheila J. Barton, Sarah El-Heis, Elizabeth H. Tham, Keith M. Godfrey, Shiao-Yng Chan, Wayne S. Cutfield, NiPPeR Study Group, Aristea Binea, Mary Cavanagh, Hsin Fang Chan, Yap Seng Chong, Paula Costello, Vanessa Cox, Judith Hammond, Nicholas C. Harvey, Soo Min Han, Mrunalini Jagtap, Justin M. O’Sullivan, Irma Silva-Zolezzi, Phil Titcombe, Mark Vickers, Gladys Woon

**Affiliations:** 1 Liggins Institute, University of Auckland, Auckland, New Zealand; 2 Department of Paediatrics: Child and Youth Health, Faculty of Medical and Health Sciences, University of Auckland, Auckland, New Zealand; 3 Environmental-Occupational Health Sciences and Non-Communicable Diseases Research Group, Research Institute for Health Sciences, Chiang Mai University, Chiang Mai, Thailand; 4 Department of Women’s and Children’s Health, Uppsala University, Uppsala, Sweden; 5 School of Chemistry and Molecular Biosciences, University of Queensland, Brisbane, Australia; 6 Department of Medicine and Department of General Practice and Primary Health Care, University of Auckland, Auckland, New Zealand; 7 Nestlé Institute of Health Sciences, Nestlé Research, Société Des Produits Nestlé S.A., Lausanne, Switzerland; 8 Singapore Institute for Clinical Sciences, Agency for Science, Technology and Research (A*STAR), Singapore, Singapore; 9 Human Potential Translational Research Programme, Yong Loo Lin School of Medicine, National University of Singapore, Singapore, Singapore; 10 MRC Lifecourse Epidemiology Centre, University of Southampton, Southampton, UK; 11 NIHR Southampton Biomedical Research Centre, University of Southampton and University Hospital Southampton NHS Foundation Trust, Southampton, UK; 12 Department of Obstetrics and Gynaecology, National University of Singapore, Singapore, Singapore; 13 A Better Start—National Science Challenge, University of Auckland, Auckland, New Zealand

**Keywords:** Adiposity, Bioelectrical impedance, Body weight, Randomized controlled trial

## Abstract

**Background:**

In a multinational randomized controlled trial, we previously showed that maternal supplementation with *myo*-inositol, probiotics, and micronutrients was associated with reduced incidence of rapid infant weight gain and high body mass index (BMI) at two years among offspring. It was unclear whether these differences in weight gain and body mass were due to reduced adiposity. Therefore, we aimed to determine whether there were any differences in body composition.

**Methods:**

Body composition was measured using bioelectrical impedance spectroscopy at six weeks, six months, one year, and two years among offspring born to mothers who received a nutritional intervention (*n* = 268) or control (*n* = 264) supplement preconception and during pregnancy.

**Results:**

There were no group-level differences in body composition, except at two years, when fat-free mass was greater among control offspring [adjusted mean difference (aMD) 0.14 kg, 95% confidence interval (CI) 0.03, 0.25, *P* = 0.012]. However, there were no differences in mean percentage fat mass (%FM) at any time. In both groups, rapid weight gain [Δ weight > 0.67 standard deviation (SD) from birth to one year] was associated with greater %FM (aMD 2.0% at six months, 2.0% at one year, 1.4% at two years) compared with those who did not have rapid weight gain. Likewise, high BMI (≥ 95 percentile) at two years was associated with greater %FM (aMD 2.5%).

**Conclusions:**

A maternal nutritional intervention did not lead to differences in average offspring body composition in the first two years of life. However, fewer offspring from the supplemented group experienced rapid weight gain and high BMI, characterized by greater %FM.

**Graphical abstract:**

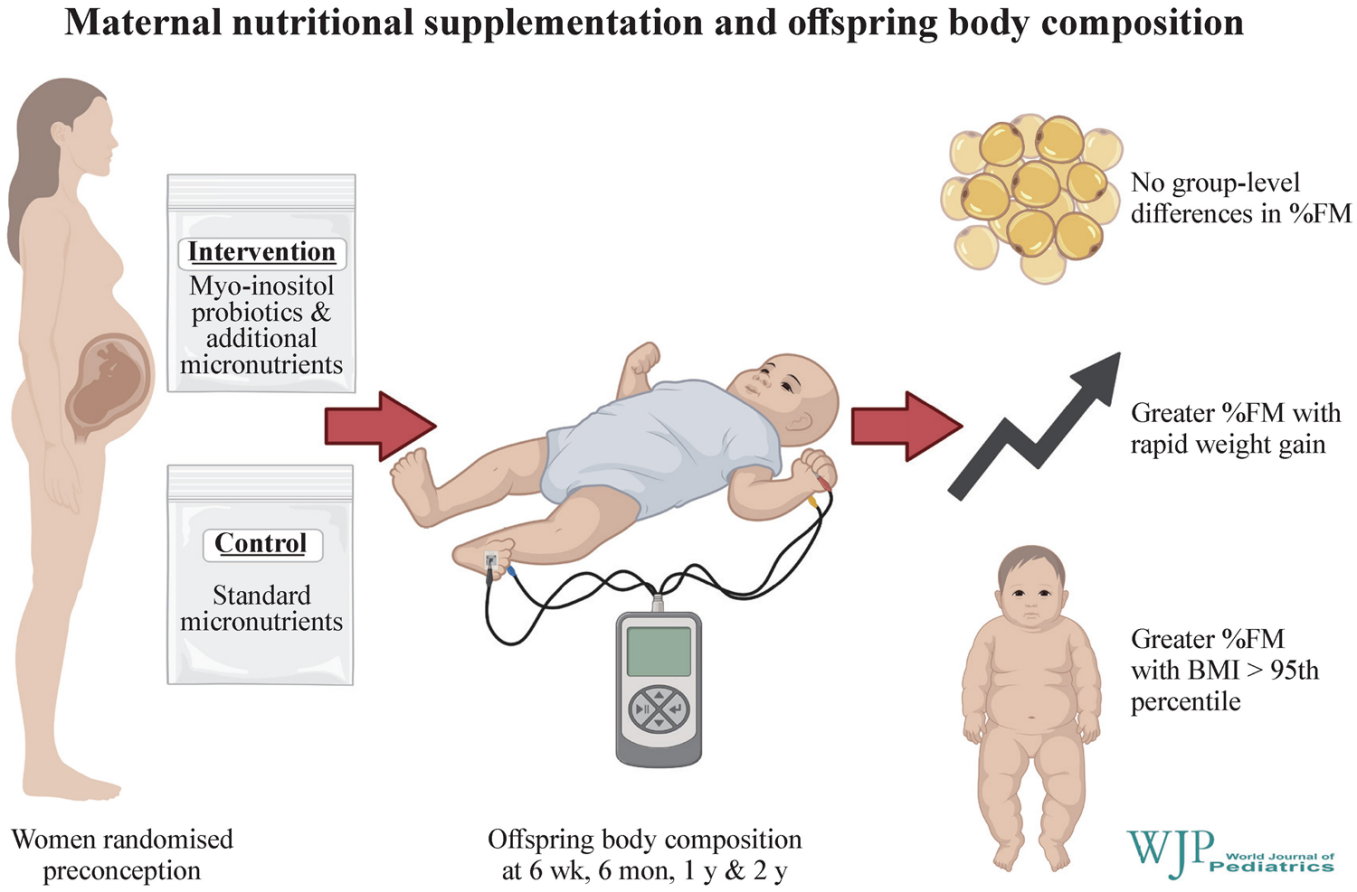

**Supplementary Information:**

The online version contains supplementary material available at 10.1007/s12519-025-00900-y.

## Introduction

Research indicates that improved maternal nutrition and healthy glycaemia during pregnancy may translate to more favourable body composition and reduced obesity risk among offspring [1–7]. For example, maternal vitamin B and D deficiencies are associated with an increased risk of offspring adiposity [1–7]. Thus, optimising maternal nutrition prior to and during pregnancy may improve offspring outcomes, including reduced adiposity.

The Nutritional Intervention Preconception and During Pregnancy to Maintain Healthy Glucose Metabolism and Offspring Health (NiPPeR) study was established to evaluate whether preconception and antenatal supplementation with *myo*-inositol, probiotics, and additional micronutrients could improve maternal and offspring outcomes [8]. While no differences were observed in the primary outcome (maternal glycaemia at 28 weeks of gestation) [9], fewer offspring born to mothers who received the intervention experienced rapid infant weight gain and had high body mass index (BMI) at two years [10]. However, as BMI cannot differentiate between fat and fat-free masses (FM and FFM), it is unclear whether these differences in body weight were due to differences in body composition.

Rapid weight gain in infancy is a known risk factor for later development of obesity and cardiometabolic disease [11–14]. Previously, accelerating weight in infancy has been associated with greater BMI and worse cardiometabolic health, such as increased cholesterol and insulin resistance at school age [15–18]. Evidence also suggests greater adiposity among school-age children who experienced rapid infant weight gain [15–19]. As obesity has been shown to track across the life course [14, 20], it is important to ascertain differences in body composition associated with rapid weight gain and high BMI in early life.

Thus, this study aimed to assess the potential effects of the NiPPeR maternal preconception and pregnancy nutritional intervention on offspring body composition in the first two years of life using previously published bioelectrical impedance spectroscopy (BIS) prediction equations.

## Methods

### Participants

Participants were offspring born to mothers participating in the NiPPeR study with at least one BIS measurement in the first two years. The NiPPeR study is a multinational randomised controlled trial with three centres: Southampton, UK; Singapore; and Auckland, New Zealand [8]. The NiPPeR trial was registered on 16 July 2015 (ClinicalTrials.gov NCT02509988; Universal Trial Number U1111-1171-8056) and was conducted according to the guidelines in the Declaration of Helsinki. Written informed consent was obtained from the mothers of the study participants. Local ethics boards approved all procedures: in the UK, the Health Research Authority National Research Ethics Service Committee South Central Research Ethics Committee (15/SC/0142); in Singapore, the National Healthcare Group Domain Specific Review Board Singapore (2015/00205); and in New Zealand, the Health and Disability Ethics Committee New Zealand (15/NTA/21).

### Intervention

Women in the NiPPeR trial were randomly allocated in a 1:1 ratio to receive a twice-daily intervention or control nutritional supplement. Both the intervention and control supplement contained folic acid (400 μg/day), iron (12 mg/day), calcium (150 mg/day), iodine (150 μg/day), and *β*-carotene (720 μg/day). The intervention additionally contained *myo*-inositol (4 g/day), vitamin D (10 μg/day), riboflavin (1.8 mg/day), vitamin B6 (2.6 mg/day), vitamin B12 (5.2 μg/day), zinc (10 mg/day), and probiotics [*Lactobacillus rhamnosus* NCC 4007 (CGMCC 1.3724) and *Bifidobacterium animalis* species lactis NCC 2818 (CNCM I-3446)]. The rationale for the composition of the nutritional intervention has previously been described [8]. The nutritional intervention started in the preconception period and was taken throughout pregnancy, with 96.6% of participants having good adherence to the study protocol [9].

### Anthropometry

Birthweight was obtained from hospital records, and subsequent weight measurements in infancy were obtained at the nearest 1 g using the SECA 376 scales (SECA, Hamburg, Germany). At two years, weight was obtained to the nearest 100 g using SECA 899 scales. Crown-heel length was measured to the nearest 0.1 cm until one year using a neonatometer (Holtain Ltd, Crymych, UK) or infantometer (Holtain Ltd). At two years, standing height was measured to the nearest 0.1 cm using a SECA 213 stadiometer.

### Bioelectrical impedance spectroscopy

Bioelectrical impedance techniques are inexpensive, portable, and widely available, with the added benefit that they can be used from birth and throughout the life course [21]. Nonetheless, the technique requires careful standardisation and the use of a population-specific equation or algorithm and coefficients, few of which are suitable for use in healthy infant populations [21]. Previously, we developed BIS prediction equations based on air displacement plethysmography among our cohort, which could predict FFM with biases < 100 g (< 2% of mean FFM) when validated [22].

BIS measurements were obtained at six weeks, six months, one year, and two years. Measurements were made using an ImpediMed SFB7 (ImpediMed, Brisbane, Australia), which measures bioimpedance parameters over a frequency range of 3 to 1000 kHz. Detailed methodology has previously been reported [23]. Briefly, ImpediMed single-tab Ag–AgCl gel electrodes (25 × 23 mm) were used to attach sense leads to the left or right dorsum wrist and ankle, with source leads attached to the palm at the metacarpal heads and the sole at the metatarsal heads (on the same side of the body). Infants were measured supine on a non-conductive examination bed, with legs apart and arms by their sides separated from the torso.

The SFB7 produces estimates of body composition using Cole modelling and mixture theory prediction [23, 24]; however, we have previously shown this to be inaccurate in infancy [22]. Therefore, measured resistance at 50 kHz (R_50_) was used to estimate FFM (kg) with previously published prediction equations developed among our cohort at six weeks and six months derived from air displacement plethysmography (PEA POD) measurements [22]:

- six-week males: FFM = 0.23 + 0.65W + 0.10L^2^/R_50_.
- six-week females: FFM = 0.60 + 0.58W + 0.08L^2^/R_50_.
- six-month males: FFM = 1.26 + 0.46W + 0.14L^2^/R_50_.
- six-month females: FFM = 1.40 + 0.36W + 0.21L^2^/R_50_.

Abbreviations: *W* scale weight (kg), *L* length (cm), *R*_50_ resistance at 50 kHz.

FM (kg) was calculated as the difference between scale weight and estimated FFM, with fat mass percentage (%FM) subsequently being calculated:

$$\begin{aligned} {\text{FM}} & = {\text{W}} - {\text{FFM}} \\ {\text{\% FM}} & = {\text{[FM/(FM + FFM)]}} \times {\text{100}} \\ \end{aligned}$$

The six-month equations were also applied at one and two years. As bioimpedance equations require a suitable age match, we also derived body composition estimates from bioimpedance measurements using two alternative equations: those developed among our New Zealand cohort at 3.5 years using dual-energy X-ray absorptiometry (DXA; GE Lunar iDXA) as the reference standard [25]; and those developed by Rush et al. [26] among New Zealand two-year-olds using DXA (GE Lunar Prodigy).

### Data analyses

Between-group differences in body composition were analysed using linear mixed models with a repeated measures design. The outcomes assessed included the primary outcome of %FM, as well as FM, FFM and their indexes (FMI and FFMI, respectively), which account for differences in stature (as kg/m^2^) [27, 28]. Models assessing the effect of the intervention compared with control were adjusted for the study site (UK/Singapore/New Zealand), as well as visit (six weeks, six months, one year, two years), a visit*randomisation group interaction term, parity (nulliparous/parous), maternal smoking during pregnancy (none/passive or active), maternal pre-pregnancy BMI, gestational age at birth, and infant sex (male/female). Additional models were run adjusted for maternal self-reported ethnicity (White Caucasian/Chinese/South Asian/Malay/other) due to previously reported differences in body composition from birth according to ethnicity [29, 30]; however, its inclusion did not notably improve model fit and therefore was disregarded. Changes (∆) in body composition parameters (%FM, FM, FFM, FMI, and FFMI) from six weeks to two years were assessed through general linear models adjusting for the aforementioned variables, using the exact age at the six-week visit and the time interval between the six-week and two-year visit instead of the ordinal visit variable.

The levels of agreement between the bioimpedance equations (NiPPeR PEA POD [22], NiPPeR DXA [25], and Rush et al. [26]) were evaluated by assessing the percentage of infants assigned to the same tertile of body composition for %FM, FM, and FFM at one and two years of age, and by Fleiss’ kappa statistic [31]. In addition, analyses were repeated considering the alternative equations, and the estimated between-group differences were subsequently compared.

As reported previously, the intervention was associated with a reduction in the incidence of rapid weight gain, defined as a weight increase > 0.67 standard deviation (SD) from birth to one year and by an increase > 1.34 SD from birth to two years [10]. Rapid weight gain in infancy and early childhood is associated with an increased risk of obesity [13]. Therefore, we also examined potential differences in body composition between children who did or did not experience rapid weight gain, irrespective of whether their mothers received the intervention (i.e., adjusting for randomization group and the aforementioned confounding factors in our models). Likewise, we examined differences in body composition between those with and without a BMI > 95th percentile (i.e., obesity) at two years [10].

Statistical analyses were run in SAS version 9.4 (SAS Institute, Cary, North Carolina, USA) or R version 4.0.3 (R Foundation for Statistical Computing, Vienna, Austria). Figures were created with GraphPad Prism version 8.2.1 (GraphPad Software, San Diego, California, USA). Baseline characteristics were compared between randomisation groups using independent samples *t*-tests or Chi-squared tests. Continuous outcomes are reported as the least squares means (i.e., adjusted means) with respective 95% confidence intervals (CI) and adjusted mean differences (aMD) and 95% CI, where relevant. All tests were two-tailed, and statistical significance was set at *P* < 0.05.

## Results

### Study participants

Characteristics of the included participants are detailed in Table 1. Baseline characteristics were similar in the two randomisation groups, except fewer mothers in the intervention group were nulliparous, and intervention offspring were less likely to be exposed to passive smoking in utero. The number of BIS measurements available for analysis at six weeks, six months, one year, and two years were 369, 429, 408, and 362, respectively (Supplementary Fig. 1).

**Table 1:** Characteristics of the study participants

Variables	Intervention (*n* = 268)	Control (*n* = 264)
Study site		
United Kingdom	82 (30.6%)	76 (28.8%)
Singapore	79 (29.5%)	77 (29.2%)
New Zealand	107 (39.9%)	111 (42.0%)
Maternal ethnicity^a^		
White Caucasian	161 (60.1%)	142 (57.5%)
Chinese	67 (25.0%)	69 (26.1%)
South Asian	13 (4.8%)	12 (4.6%)
Malay	11 (4.1%)	12 (4.6%)
Others	16 (6.0%)	19 (7.2%)
Maternal BMI (kg/m^2^)	24.5 ± 5.1	25.1 ± 5.7
Maternal height (cm)	164.6 ± 6.8	163.9 ± 7.0
Parity^b^		
Nulliparous	156 (58.2%)	186 (70.4%)
Parous	112 (41.8%)	78 (29.6%)
Maternal smoking during pregnancy		
None	238 (88.8%)	215 (81.4%)
Passive smoking	22 (8.2%)	42 (15.9%)
Active smoking	8 (3.0%)	7 (2.7%)
Infant sex		
Male	130 (48.5%)	118 (44.7%)
Female	138 (51.5%)	146 (55.3%)
Gestational age (wk)^c^	39.4 ± 1.4	39.3 ± 1.6
Preterm	13 (4.9%)	22 (8.3%)
Term	253 (94.4%)	240 (90.9%)
Post-term	2 (0.7%)	2 (0.8%)
Birthweight (g)	3364 ± 510	3316 ± 531
Birthweight SDS^d^	− 0.01 ± 0.92	− 0.05 ± 0.94
SGA	21 (7.8%)	20 (7.6%)
AGA	228 (85.1%)	223 (84.5%)
LGA	19 (7.1%)	21 (8.0%)
Any breast-feeding		
Yes	259 (96.6%)	260 (98.5%)
No	9 (3.4%)	4 (1.5%)
Never exclusively breastfed^f^		
Yes	156 (58.4%)	143 (54.8%)
No	111 (41.6%)	118 (45.2%)
Exclusive breastfeeding duration (wk)^e,f^	8.0 ± 10.0	7.5 ± 9.9
Missing	1	3
Any breastfeeding duration (wk)^f^	40.2 ± 17.1	37.4 ± 19.3

Data are mean ± SD or *n* (%). ^a^ South Asian includes Indian, Pakistani, and Bangladeshi mothers; Other includes mothers of mixed, Black, or Polynesian ethnicity; ^b^ Multiparous includes mothers with one or more births > 24 weeks of gestation; ^c^ Preterm is defined as birth prior to 37^0/7^ weeks of gestation, term as birth between 37^0/7^ and 41^6/7^ weeks of completed gestation, and post-term as birth at or beyond 42^0/7^ weeks of completed gestation; ^d^ Calculated using the UK–WHO reference; SGA is defined as birth weight < 10th percentile (− 1.282 SD) and LGA as > 90th percentile (1.282 SD); ^e^ Exclusive breastfeeding is defined as feeding the infant only breast milk, with no intake of water, formula, or other liquid or solid food, except for oral rehydration solution or drops/syrups of vitamins, minerals, or medicines; ^f^ Participants who were never breastfed or not exclusively breastfed were assigned a value of 0

*SGA* small-for-gestational-age, *AGA* appropriate-for-gestational-age, *LGA* large-for-gestational-age, *BMI* body mass index, *SD* standard deviation, *SDS* standard deviation score, *WHO* World Health Organization

### Comparison of bioimpedance equations

There was moderate to strong agreement between the three bioimpedance equations (six-month PEA POD, 3.5-year DXA, and Rush equations; κ = 0.62–0.82) [32], with > 60% of offspring assigned to the same tertile of body composition (Supplementary Table 1). Estimated between-group differences from analyses run considering the alternative equations are reported in Supplementary Table 2. Given previously reported marked differences in PEA POD and DXA estimates of body composition [33, 34], we report results from analyses conducted using the PEA POD BIS equations so that the same reference standard is used at all time points.

### Body composition by visit

**Fig. 1:**
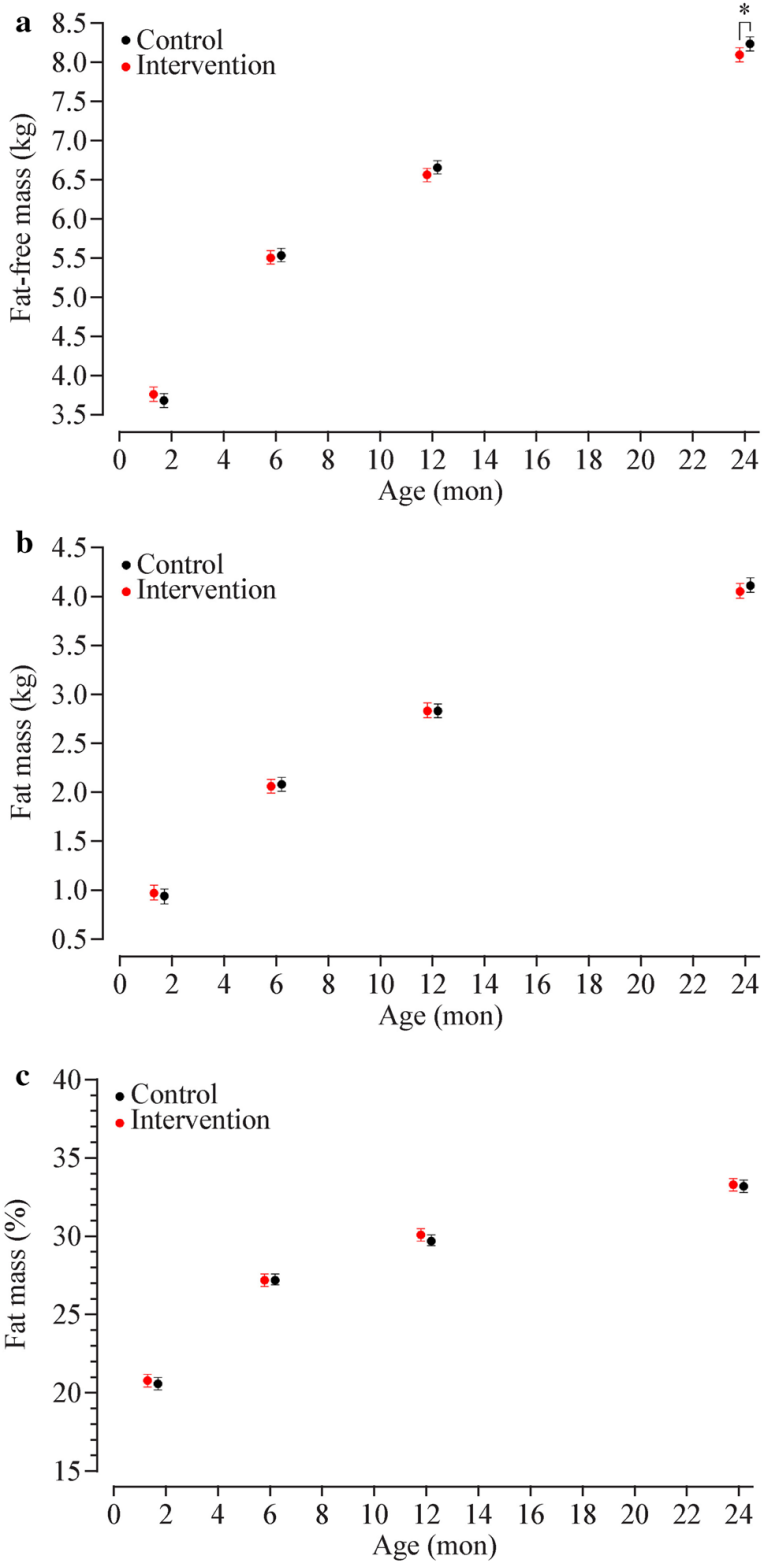
Body composition in the first two years of life in the intervention (red) and control (black) offspring. Data are the least squares means (i.e., adjusted means) and 95% confidence intervals for **a** fat-free mass (kg), **b** fat mass (kg), and **c** fat mass (%), derived from linear mixed models based on repeated measures. *P < 0.05 for a difference between groups at a given age

**Fig. 2:**
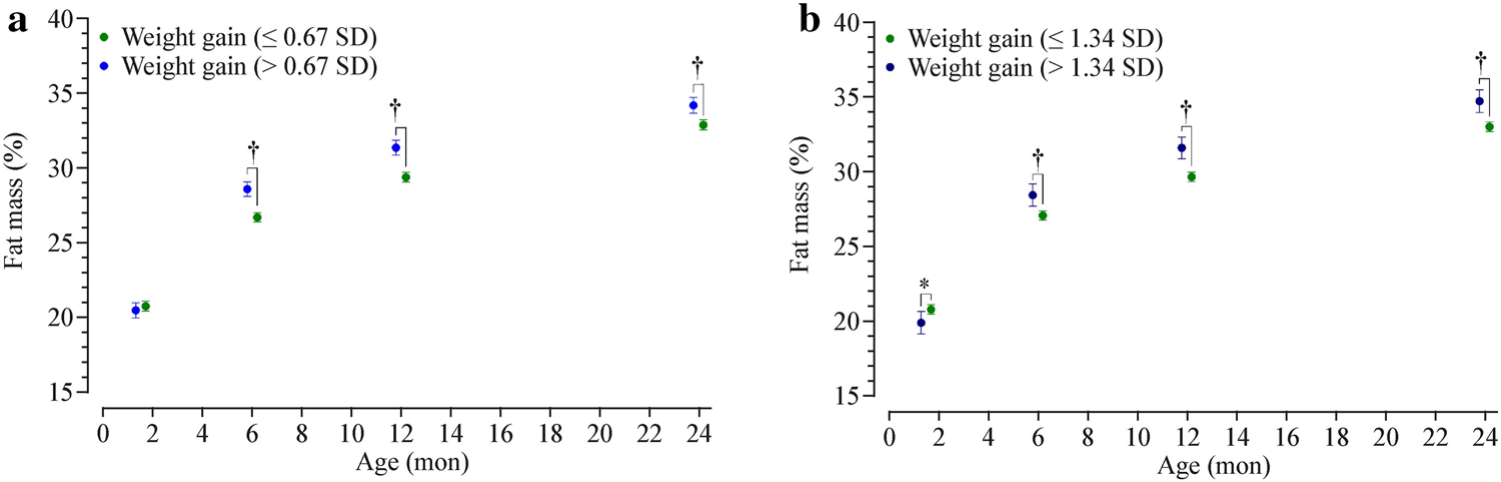
Fat mass percentage in the offspring in the first two years of life. Panels represent: **a** children who experienced rapid weight gain in the first year of life (> 0.67 SD; light blue) and those who did not (≤ 0.67 SD; green); and *b* children who experienced sustained rapid weight gain in the first two years of life (> 1.34 SD; dark blue) and those who did not (≤ 1.34 SD; green). Data are the least squares means (i.e., adjusted means) and 95% confidence intervals at each visit derived from linear mixed models based on repeated measures. *P < 0.05 and †*P* < 0.001 for a difference between the two groups at a given age. *SD* standard deviation

Analyses of bioimpedance data showed that FFM was similar in the control and intervention groups except at two years, when there was greater FFM among control offspring [aMD 0.14 kg (95% CI: 0.03, 0.25), *P* = 0.012; Fig. 1a]. This difference attenuated slightly after accounting for stature [i.e. FFMI, aMD 0.17 kg/m^2^ (95% CI: 0.02, 0.32), *P* = 0.030; Fig. 2a]. However, at two years, %FM was not different between groups [aMD − 0.1% (95% CI: − 0.6, 0.4), *P* = 0.71; Fig. 1c]. Results were not different when sensitivity analyses were run including only term-born infants (Supplementary Fig. 2).

### Changes in body composition

Analyses of the change in body composition parameters revealed no differences in FM (%, kg, or kg/m^2^) from six weeks to two years (Table 2). However, a lower increase in FFM was observed among intervention offsprings [aMD − 0.18 kg (95% CI − 0.34, − 0.03), *P* = 0.022], but this difference attenuated in analyses of FFMI that accounted for differences in stature [aMD 0.16 kg/m^2^ (95% CI − 0.03, 0.36), *P* = 0.10; Table 2]. Results from sensitivity analyses on term-born infants were unchanged (Supplementary Table 3).

**Table 2:** Changes in body composition from six weeks to two years among NiPPeR offspring, estimated by BIS

Parameters	Intervention	Control	aMD	*P*
*n*	128	121		
FM (%)	12.2 (11.5, 12.8)	11.8 (11.2, 12.5)	0.3 (− 0.4, 1.0)	0.35
FM (kg)	2.99 (2.85, 3.13)	3.02 (2.88, 3.16)	− 0.03 (− 0.19, 0.12)	0.69
FFM (kg)	4.26 (4.12, 4.40)	4.44 (4.30, 4.58)	− 0.18 (− 0.34, -0.03)	**0.022**
FMI (kg/m^2^)	2.31 (2.13, 2.48)	2.30 (2.13, 2.47)	0.01 (-0.19, 0.20)	0.94
FFMI (kg/m^2^)	− 0.91 (− 1.09, − 0.73)	− 0.74 (− 0.92, − 0.57)	− 0.16 (− 0.36, 0.03)	0.10

The statistically significant *P*-value (at *P* < 0.05) is shown in bold

Data are estimated marginal means and respective 95% confidence intervals from general linear models. Models were adjusted for randomisation group (intervention/control), exact age at the six-week visit, time interval between the six-week and two-year visit, study site (UK/Singapore/New Zealand), infant sex (male/female), parity (multiparous/nulliparous), maternal smoking during pregnancy (none/active or passive), maternal pre-pregnancy BMI, and gestational age. *aMD* adjusted mean difference, *FFM* fat-free mass, *FFMI* fat-free mass index, *FM* fat mass, *FMI* fat mass index, *BIS* bioelectrical impedance spectroscopy, *NiPPeR* Nutritional Intervention Preconception and During Pregnancy to Maintain Healthy Glucose Metabolism and Offspring Health

### Rapid weight gain and body composition

**Fig. 3:**
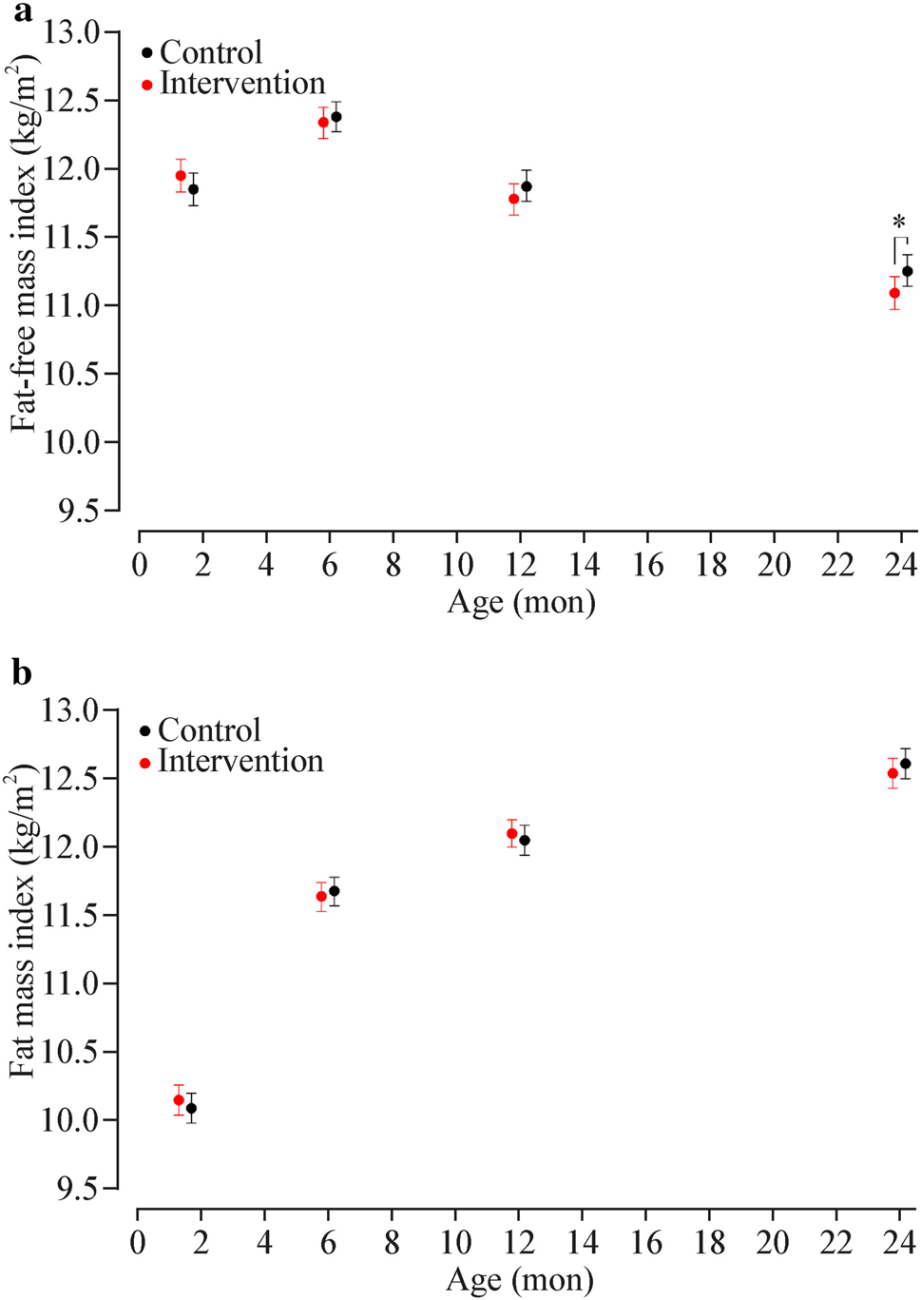
Indices of body composition in the first two years of life in the intervention (red) and control (black) offspring. Data are the least squares means (i.e., adjusted means) and 95% confidence intervals for **a** fat-free mass index (kg/m^2^) and **b** fat mass index (kg/m^2^), derived from linear mixed models based on repeated measures. *P < 0.05 for a difference between groups at a given age

Data on rapid weight gain from birth to one year were available for 512 children (96% of participants) and from birth to two years for 488 children (92%), with the percentage of infants who experienced rapid weight gain in each randomisation group detailed in Table 3. Irrespective of any intervention effects, children who experienced rapid weight gain (> 0.67 SD) in infancy had similar body composition at six weeks, but increased adiposity at six months, one year, and two years, with %FM 2.0%, 2.0%, and 1.4% higher, respectively, than those without rapid weight gain (Fig. 3a). Children who experienced sustained rapid weight gain (i.e., > 1.34 SD from birth to two years of age) had moderately lower %FM at six weeks (− 0.9%) but greater adiposity at subsequent visits (1.4%, 1.9%, and 1.7%, respectively) (Fig. 3b).

**Table 3:** Proportion of children born to mothers in the intervention and control groups who experienced rapid weight gain in the first two years of life

Categories	Intervention	Control	Total
Number	268	264	532
Rapid weight gain > 0.67 SD from birth to 1 y			
Yes	58 (22.1%)	79 (31.6%)	137 (26.8%)
No	204 (77.9%)	171 (68.4%)	375 (73.2%)
Missing	6	14	20
Rapid weight gain > 1.34 SD from birth to 2 y			
Yes	19 (7.9%)	41 (16.7%)	60 (12.3%)
No	223 (92.2%)	205 (83.3%)	428 (87.7%)
Missing	26	18	44

Data are *n* (%). *SD* standard deviation

### High body mass index and body composition

BMI and BIS data at two years were available for 362 children (68% of participants). Forty-five (12.4%) children had a BMI > 95th percentile at two years [9.4% (*n* = 17/181) intervention vs. 15.5% (*n* = 28/181) control; *P* = 0.08]. Children with BMI > 95th percentile at two years had %FM 2.5% greater than those with a BMI ≤ 95th percentile [aMD: 2.5% (95% CI: 1.8, 3.2), *P* < 0.001].

## Discussion

Previously, among the offspring cohort of the NiPPeR study, we reported less rapid weight gain in the first two years of life and high BMI at two years among children whose mothers received a nutritional intervention containing *myo*-inositol, probiotics, and additional micronutrients prior to and during pregnancy [10]. Here, we observed lower FFM among intervention offspring at two years. However, observed differences were marginal (− 0.14 kg), and %FM was not different between the randomisation groups. Thus, overall, the intervention did not lead to robust differences in average body composition detectable by BIS in the first two years of life. Nonetheless, irrespective of the intervention effects (i.e., when these were accounted for in our analyses), children who experienced rapid infant or early childhood weight gain or high BMI at two years had greater levels of adiposity.

While no other studies have assessed the effects of the specific combination of nutrients contained within the NiPPeR intervention, previous studies of antenatal supplementation with multiple micronutrients have found no impact on offspring anthropometry or body composition in childhood [35]. Likewise, no differences were reported in anthropometry or DXA estimates of body composition among offspring in a study where a food-based approach to maternal nutritional supplementation was used [36]. However, these studies were conducted among low- and middle-income countries and may not be directly comparable to our cohort [35, 36].

Individual nutrients within the NiPPeR intervention have been linked with offspring body composition. Maternal vitamin D insufficiency has been associated with increased offspring adiposity [1–5]. These associations may be influenced by maternal smoking [2] and maternal BMI [3, 4], with marked differences reported among smokers and mothers with overweight or obesity. However, most evidence relating maternal vitamin D and offspring body composition is observational. Even so, among offspring whose mothers were randomised to receive antenatal vitamin D supplementation (25 µg/day cholecalciferol) or a placebo at four years of age, differences were observed in DXA estimates of lean mass but not FM. Nonetheless, these associations attenuated to the null following adjustment for age, sex, and height or weight [37]. Maternal B-vitamin deficiencies have likewise been associated with increased offspring insulin resistance and adiposity [6, 7]. Limited experimental evidence exists, although a randomised controlled trial where mothers received vitamin B12 (2 µg/day with or without multiple micronutrients) or a placebo found no differences in neonatal anthropometry (weight, length, and head circumference) [38]. Similarly, among 729 Nepalese offspring whose mothers received a high dose of vitamin B12 (50 µg/day) or control nutritional intervention alongside standard pregnancy supplements (folic acid and calcium) from early pregnancy through to six months postpartum, there were no differences in birthweight, as well as in anthropometry (weight, length, and BMI) at six months or one year [39]. However, studies are ongoing, and further results related to infant growth and body composition are yet to be published [40, 41]. Zinc supplementation in infancy and childhood has been widely studied and is associated with improved linear growth and weight gain among deficient populations [42]. In contrast, the results of maternal supplementation intervention studies are less clear and likely involve the effects of multiple nutrients [43]. *Myo*-inositol and probiotics are also hypothesised to have protective effects against offspring adiposity, but research is preliminary, and further investigation is required [44, 45].

While we did not observe an intervention effect on average body composition in the first two years, we previously reported lower rates of rapid weight gain and high BMI among intervention offspring [10]. Here, we show that offspring who experienced rapid weight gain had greater %FM, irrespective of any potential intervention effects. Several studies have linked accelerated weight gain in infancy with increased adiposity and risk of obesity in later childhood [15–18, 46]. However, few have examined longitudinal data on the associations between rapid weight gain and infant body composition [17, 19]. These studies have similarly reported rapid weight gain largely attributable to FM gain [17, 19]. While both FM and FFM track into later childhood, evidence suggests that high neonatal FFM is associated with high FFM, FM, and FFMI later in life, but not FMI [46, 47], which indicates that FM increases proportionally with the infant’s size [47]. In contrast, high FM at birth and high FM gain in infancy are associated with increased proportional FM (i.e. FMI) but not FFM in later childhood [46, 47]. Previous evidence suggests that FFM is more strongly associated with linear growth [48, 49], while factors such as maternal pre-pregnancy BMI and infant feeding have a greater influence on FM [49–52]. Our findings support the notion that early rapid weight gain is largely attributable to FM gain, which in turn can lead to excessive adiposity and poorer metabolic health in the long term.

Strengths of the current study include data collection from multiple time points in the first two years of life among a multi-ethnic cohort of infants in three countries. All tools used to assess body composition have strengths and limitations; few can be used across the age span [53]. While bioimpedance techniques can measure body composition across the age span, they require population-specific equations or coefficients. In this study, we were limited by the availability of equations suitable for use beyond six months of age [21]. Still, our findings were comparable when we applied alternative equations to our population at one and two years [25, 26].

Obtaining body composition estimates in infancy and early childhood is challenging. While a multi-component reference model can provide further information about inter-individual variation in body composition [53], simpler methods are required due to the time-consuming nature of the technique. However, the accuracy of the PEA POD [34, 54], DXA [34], and BIS [55] remains debatable. Another limitation of this study was the relatively short duration of follow-up. High adiposity in early life has been shown to track into later childhood [56]; however, there are many pathways into and out of obesity [14], and numerous factors may influence obesity [12]. In a large retrospective study of over 50,000 individuals, most children with obesity (identified using BMI) were found to continue to have overweight or obesity in adolescence [14]. Among the same cohort, prospectively, it was found that among adolescents with obesity, the greatest acceleration in BMI was observed in early childhood [14]. Thus, it is important to ascertain whether our cohort’s reduced incidence of rapid weight gain in infancy with the intervention translates to reduced adiposity in the long term.

In conclusion, while there were no observable differences in body composition between the intervention and control groups, we previously reported that the NiPPeR intervention was associated with a reduction in the incidence of rapid weight gain in infancy and early childhood and high BMI at two years. Importantly, in this study, children in these high-risk subgroups had greater levels of adiposity. Future research should establish if body composition and cardiometabolic markers are altered among intervention offspring in later childhood and adolescence.

## Supplementary Information

Below is the link to the electronic supplementary material.

Supplementary file1 (PDF 581 KB)

## Data Availability

The datasets generated and/or analysed during the current study are not publicly available due to the participants not consenting to open access data sharing and this being an ongoing longitudinal study in which there will be further future analyses conducted, but are available from the corresponding author on reasonable request.
