# Supplementary material for: Infant body composition in a randomised trial of a maternal nutritional supplement during preconception and pregnancy
Source: World J Pediatr. 2025 May 14;21(4):361–71. doi: 10.1007/s12519-025-00900-y (PMC12103482; doi:10.1007/s12519-025-00900-y)

Supplementary File

Infant body composition in a randomised trial of a maternal nutritional supplement during preconception and pregnancy

J. Lyons-Reid[1], J. G. B. Derraik[2,3,4], L. C. Ward[5], T. Kenealy[1,6], B. B. Albert[1], J. M. Ramos Nieves[7], C. R. Monnard[7], M. Thway-Tint [8,9], H. Nield[10], S. J. Barton[10], S. El-Heis[10,11], E. H. Tham[8,9,12], K. M. Godfrey[10,11,#], S.-Y. Chan[8,9,12.#], W. S. Cutfield[1,13,#,*] on behalf of the NiPPeR Study Group[‡]

[1] Liggins Institute, University of Auckland, Auckland, New Zealand
[2] Department of Paediatrics: Child and Youth Health, Faculty of Medical and Health Sciences, University of Auckland, Auckland, New Zealand
[3] Environmental-Occupational Health Sciences and Non-communicable Diseases Research Group, Research Institute for Health Sciences, Chiang Mai University, Chiang Mai, Thailand
[4] Department of Women's and Children's Health, Uppsala University, Uppsala, Sweden
[5] School of Chemistry and Molecular Biosciences, University of Queensland, Brisbane, Australia
[6] Department of Medicine and Department of General Practice and Primary Health Care, University of Auckland, Auckland, New Zealand
[7] Nestlé Institute of Health Sciences, Nestlé Research, Société des Produits Nestlé S.A., Lausanne, Switzerland
[8] Singapore Institute for Clinical Sciences, Agency for Science, Technology and Research (A*STAR), Singapore
[9] Human Potential Translational Research Programme, Yong Loo Lin School of Medicine, National University of Singapore, Singapore
[10] MRC Lifecourse Epidemiology Centre, University of Southampton, Southampton, United Kingdom
[11] NIHR Southampton Biomedical Research Centre, University of Southampton and University Hospital Southampton NHS Foundation Trust, Southampton, United Kingdom
[12] Department of Obstetrics & Gynaecology, National University of Singapore, Singapore
[13] A Better Start – National Science Challenge, University of Auckland, Auckland, New Zealand

[#] Joint senior authors

[‡] The NiPPeR study group authors for the Medline citation comprises the following: Aristea Binea, Mary Cavanagh, Hsin Fang Chan, Yap Seng Chong, Paula Costello, Vanessa Cox, Judith Hammond, Nicholas C Harvey, Soo Min Han, Mrunalini Jagtap, Justin M O'Sullivan, Irma Silva-Zolezzi, Phil Titcombe, Mark Vickers, and Gladys Woon.

Corresponding author: Professor Wayne Cutfield, Liggins Institute, University of Auckland, Private Bag 92019, Auckland 1142, New Zealand. Email: w.cutfield@auckland.ac.nz; Ph: +64 9 923 4476

Supplementary Table S1

Agreement between the three bioimpedance equations (NiPPeR 6-week/6-month PEA POD equation[1], NiPPeR 3.5-year dual-energy X-ray absorptiometry (DXA) equation[2], or Rush 2-year DXA equation[3]) in body composition tertile assignment at 1 and 2 years.

	Fat mass (%)		Fat mass (kg)		Fat-free mass (kg)	
	% agreement	Kappa	% agreement	Kappa	% agreement	Kappa
**1 year**	68.5	0.682	61.7	0.616	79.5	0.795
**2 years**	66.0	0.658	65.5	0.653	81.6	0.816

Abbreviations: DXA, whole-body dual-energy X-ray absorptiometry.
Agreement in tertile assignment reported as percentage agreement and kappa statistics.
[1] PEA POD equation: developed among 145 New Zealand 6-week-olds and 206 6-month-olds using the PEA POD Infant Body Composition System as the reference standard (Lyons-Reid et al. Front Nutr 2022;9:980790).
[2] DXA equation: developed among 45 New Zealand 3.5-year-olds using a GE Lunar iDXA device as the reference standard (Lyons-Reid et al. Eur J Clin Nutr 2024;78:872-9).
[3] Rush et al. DXA equation: developed among 77 New Zealand 2-year-olds using a GE Lunar Prodigy DXA device as the reference standard (Rush et al. Eur J Clin Nutr 2013;67:214-7).

Supplementary Table S2

Comparisons of estimated between-group differences (adjusted mean differences) from 6 weeks to 2 years according to the bioimpedance equation applied (NiPPeR 6-week/6-month PEA POD equation, NiPPeR 3.5-year dual-energy X-ray absorptiometry (DXA) equation, or Rush 2-year DXA equation).

	6 weeks [1]		6 months [1]		1 year [1]		2 years [1]		Δ 6 weeks to 2 years [2]	
	aMD [3]	*p*	aMD [3]	*p*	aMD [3]	*p*	aMD [3]	*p*	aMD [3]	*p*
**Fat mass (%)**										
PEA POD eq [4]	0.2 (-0.3, 0.7)	0.46	0.0 (-0.5, 0.4)	0.88	0.4 (-0.1, 0.8)	0.14	0.1 (-0.4, 0.6)	0.71	0.3 (-0.4, 1.0)	0.35
DXA eq [5]	0.2 (-0.6, 0.9)	0.65	0.0 (-0.7, 0.6)	0.91	0.9 (0.2, 1.6)	**0.009**	0.4 (-0.3, 1.1)	0.29	0.8 (-0.4, 2.0)	0.18
Rush eq [6]	0.1 (-0.6, 0.9)	0.71	-0.1 (-0.8, 0.7)	0.87	0.7 (0.0, 1.5)	0.052	0.0 (-0.8, 0.8)	0.95	0.6 (-0.7, 2.0)	0.37
**Fat mass (kg)**										
PEA POD eq [4]	0.04 (-0.06, 0.13)	0.44	-0.02 (-0.11, 0.07)	0.63	0.00 (-0.08, 0.09)	0.92	-0.06 (-0.15, 0.04)	0.22	-0.03 (-0.19, 0.12)	0.69
DXA eq [5]	0.03 (-0.08, 0.13)	0.61	-0.03 (-0.12, 0.07)	0.61	0.06 (-0.04, 0.16)	0.23	-0.01 (-0.11, 0.10)	0.89	0.04 (-0.15, 0.22)	0.68
Rush eq [6]	0.03 (-0.08, 0.13)	0.63	-0.03 (-0.12, 0.07)	0.59	0.04 (-0.05, 0.14)	0.38	-0.04 (-0.15, 0.06)	0.43	0.03 (-0.17, 0.22)	0.79
**Fat-free mass (kg)**										
PEA POD eq [4]	0.08 (-0.03, 0.19)	0.14	-0.03 (-0.13, 0.07)	0.55	-0.10 (-0.20, 0.01)	0.064	-0.14 (-0.25, -0.03)	**0.012**	-0.18 (-0.34, -0.03)	**0.022**
DXA eq [5]	0.08 (-0.05, 0.21)	0.23	-0.03 (-0.15, 0.09)	0.64	-0.16 (-0.28, -0.03)	**0.012**	-0.18 (-0.31, -0.04)	**0.009**	-0.25 (-0.45, -0.05)	**0.014**
Rush eq [6]	0.08 (-0.05, 0.22)	0.22	-0.03 (-0.15, 0.10)	0.66	-0.14 (-0.27, -0.02)	**0.028**	-0.14 (-0.28, -0.01)	**0.034**	-0.24 (-0.44, -0.03)	**0.023**
**Fat mass index (kg/m²)**										
PEA POD eq [4]	0.06 (-0.08, 0.20)	0.38	-0.04 (-0.17, 0.09)	0.56	0.05 (-0.08, 0.18)	0.48	-0.07 (-0.21, 0.07)	0.34	0.01 (-0.19, 0.20)	0.94
DXA eq [5]	0.05 (-0.11, 0.22)	0.53	-0.04 (-0.20, 0.11)	0.58	0.15 (-0.01, 0.30)	0.065	0.00 (-0.17, 0.16)	0.99	0.10 (-0.15, 0.34)	0.45
Rush eq [6]	0.05 (-0.12, 0.22)	0.55	-0.05 (-0.21, 0.11)	0.56	0.11 (-0.05, 0.27)	0.19	-0.05 (-0.22, 0.12)	0.59	0.06 (-0.20, 0.33)	0.64
**Fat-free mass index (kg/m²)**										
PEA POD eq [4]	0.10 (-0.05, 0.25)	0.18	-0.05 (-0.19, 0.09)	0.51	-0.10 (-0.24, 0.05)	0.18	-0.17 (-0.32, -0.02)	**0.030**	-0.16 (-0.36, 0.03)	0.10
DXA eq [5]	0.10 (-0.07, 0.27)	0.25	-0.04 (-0.21, 0.12)	0.59	-0.20 (-0.37, -0.04)	**0.016**	-0.23 (-0.40, -0.05)	**0.011**	-0.25 (-0.48, -0.01)	**0.038**
Rush eq [6]	0.10 (-0.06, 0.26)	0.20	-0.04 (-0.19, 0.10)	0.57	-0.17 (-0.32, -0.02)	**0.027**	-0.18 (-0.34, -0.02)	**0.024**	-0.22 (-0.44, 0.00)	**0.049**

Abbreviations: aMD, adjusted mean difference; DXA, whole-body dual-energy X-ray absorptiometry.
[1] Data are adjusted mean differences and respective 95% confidence intervals from linear mixed models with a repeated measures design. Models are adjusted for randomisation group (intervention/control), visit*randomisation group interaction term, study site (UK/Singapore/New Zealand), infant sex (male/female), parity (multiparous/nulliparous), maternal smoking during pregnancy (none/active or passive), maternal pre-pregnancy BMI, and gestational age.
[2] Data are adjusted mean differences and respective 95% confidence intervals from general linear models. Models are adjusted for randomisation group (intervention/control), exact age at the 6-week visit, time interval between the 6-week and 2-year visit, study site (UK/Singapore/New Zealand), infant sex (male/female), parity (multiparous/nulliparous), maternal smoking during pregnancy (none/active or passive), maternal pre-pregnancy BMI, and gestational age. Statistically significant comparisons ($p<0.05$) are shown in bold.
[3] Adjusted mean difference (Intervention – Control).
[4] PEA POD equation: developed among 145 New Zealand 6-week-olds and 206 6-month-olds using the PEA POD Infant Body Composition System as the reference standard (Lyons-Reid et al. Front Nutr 2022; 9:980790).
[5] DXA equation: developed among 45 New Zealand 3.5-year-olds using a GE Lunar iDXA device as the reference standard (Lyons-Reid et al. Eur J Clin Nutr 2024;78:872-9).
[6] Rush et al. DXA equation: developed among 77 New Zealand 2-year-olds using a GE Lunar Prodigy DXA device as the reference standard (Rush et al. Eur J Clin Nutr 2013; 67:214-7).

Supplementary Table S3

Changes in body composition from 6 weeks to 2 years among NiPPeR offspring born at term, estimated by bioelectrical impedance spectroscopy (BIS).

Parameter	Intervention	Control	aMD	*p*
n	121	109		
**FM (%)**	12.0 (11.4, 12.7)	11.8 (11.2, 12.5)	0.2 (-0.5, 1.0)	0.51
**FM (kg)**	2.97 (2.82, 3.12)	3.02 (2.88, 3.17)	-0.05 (-0.21, 0.11)	0.53
**FFM (kg)**	4.22 (4.08, 4.37)	4.40 (4.25, 4.55)	-0.18 (-0.34, -0.02)	**0.031**
**FMI (kg/m^2)**	2.28 (2.10, 2.46)	2.30 (2.12, 2.48)	-0.02 (-0.21, 0.18)	0.88
**FFMI (kg/m^2)**	-0.91 (-1.09, -0.73)	-0.75 (-0.93, -0.58)	-0.16 (-0.35, 0.04)	0.11

Abbreviations: aMD, adjusted mean difference; FFM, fat-free mass; FFMI, fat-free mass index; FM, fat mass; FMI, fat mass index.
Data are estimated marginal means and respective 95% confidence intervals from general linear models. Models are adjusted for randomisation group (intervention/control), exact age at the 6-week visit, time interval between the 6-week and 2-year visit, study site (UK/Singapore/New Zealand), infant sex (male/female), parity (multiparous/nulliparous), maternal smoking during pregnancy (none/active or passive), maternal pre-pregnancy BMI, and gestational age.
Statistically significant comparisons (p<0.05) are shown in bold.

Supplementary Figure S1

CONSORT flow diagram showing the recruitment and assessments of mothers and infants throughout the NiPPeR randomized controlled trial.

Note that the post-randomization withdrawals consisted of the following: new type 2 diabetes diagnosis after randomization (*n*=40); voluntary withdrawal/loss to follow-up/other (*n*=395); did not conceive within 1 year (*n*=311); became pregnant before preconception visit 2 (*n*=117); use of assisted conception (*n*=87); product intolerance/logistics (*n*=47); or a new medical disorder (*n*=7) (Godfrey et al. Diabetes Care 2021;44:1091-9).

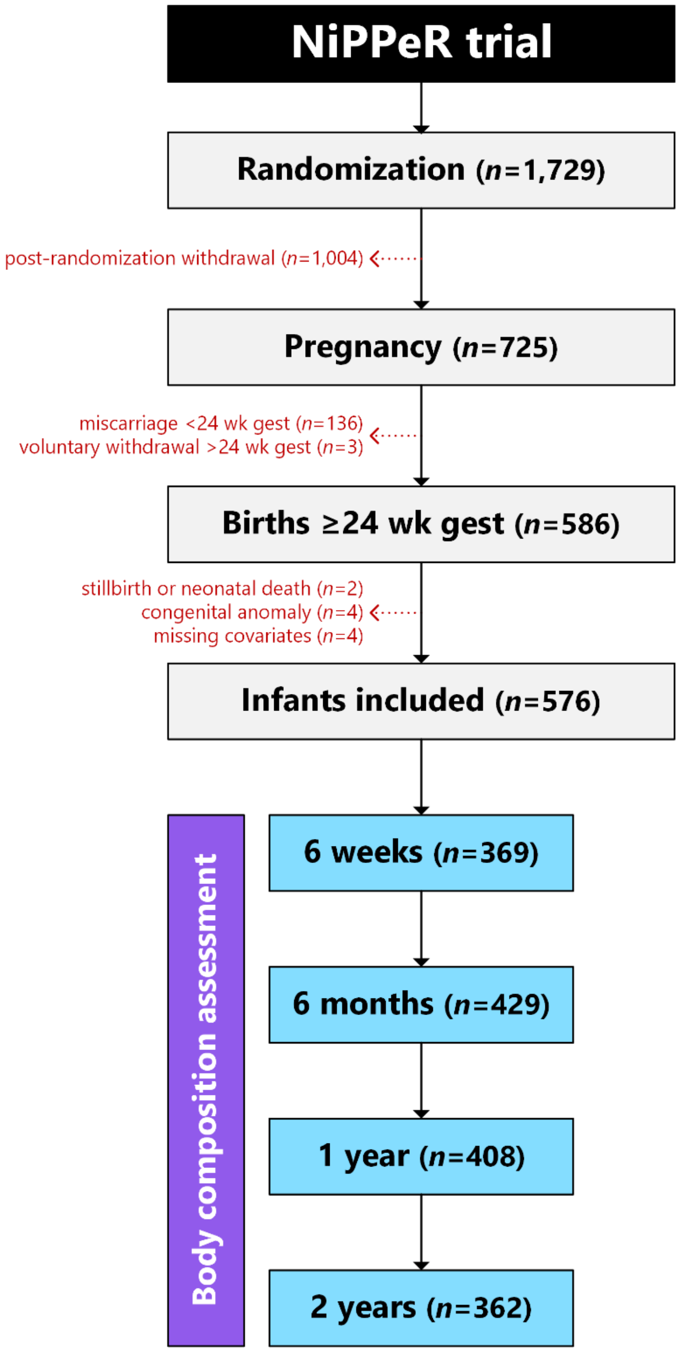

Supplementary Figure S2

Body composition in the first 2 years of life among intervention (red) and control (black) offspring born at term.

Data are the least squares means (i.e., adjusted means) and 95% confidence intervals at each visit for A) fat-free mass (kg) B) fat mass (kg), C) fat mass (%), D) fat-free mass index (kg/m^2), and E) fat mass index (kg/m^2) derived from repeated measures linear mixed models for. *p<0.05 and **p<0.01 for a difference between groups at a given age.

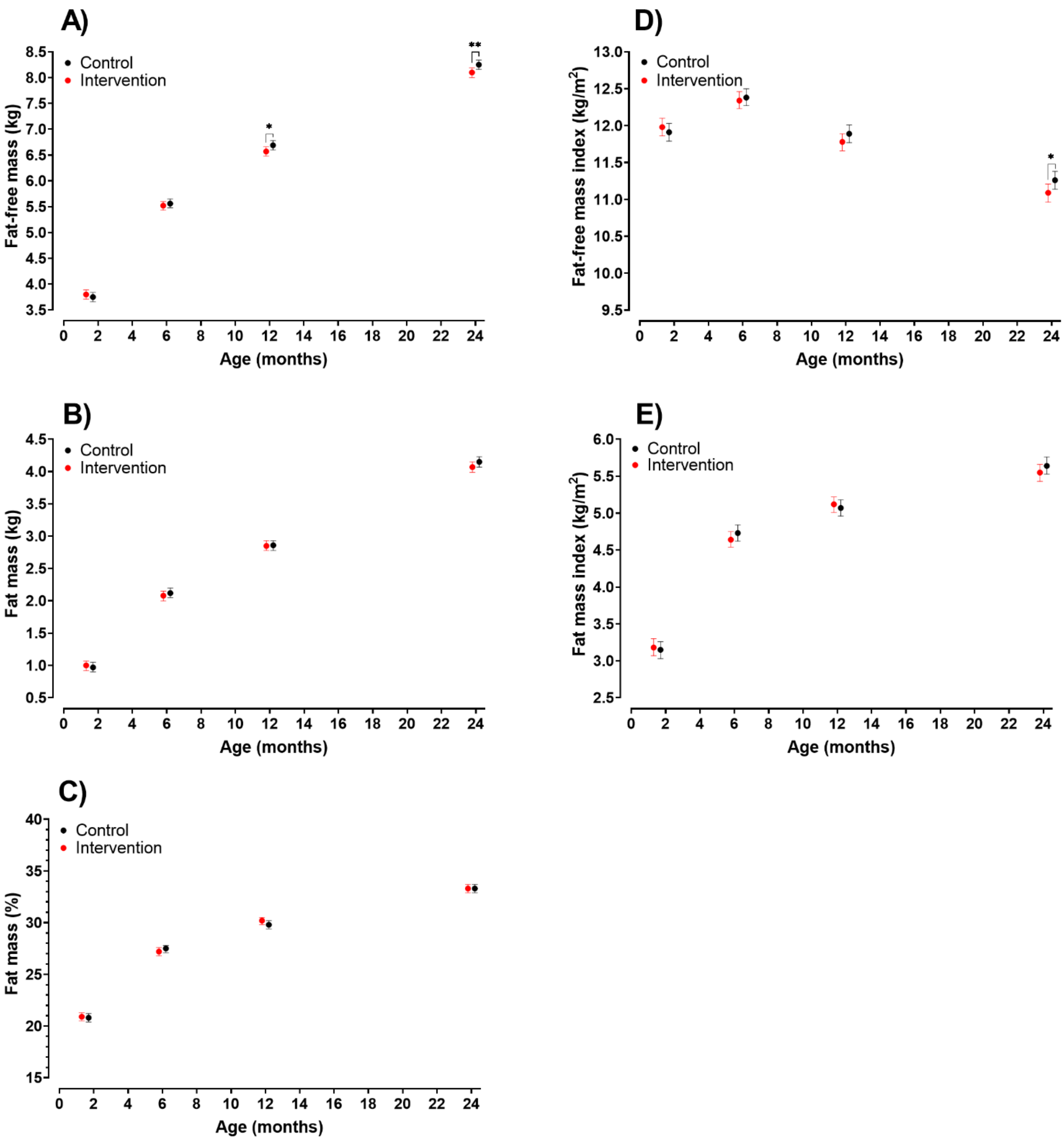

Supplement: Supplementary file 1 — Supplementary file1 (PDF 581 KB) [file 12519_2025_900_MOESM1_ESM.pdf]
